# Core Muscle Activity, Exercise Preference, and Perceived Exertion during Core Exercise with Elastic Resistance versus Machine

**DOI:** 10.1155/2015/403068

**Published:** 2015-10-18

**Authors:** Jonas Vinstrup, Emil Sundstrup, Mikkel Brandt, Markus D. Jakobsen, Joaquin Calatayud, Lars L. Andersen

**Affiliations:** ^1^National Research Centre for the Working Environment, Lersø Parkallé 105, 2100 Copenhagen Ø, Denmark; ^2^Physical Activity and Human Performance Group, SMI, Department of Health Science and Technology, Aalborg University, 9220 Aalborg, Denmark; ^3^Prevention Health Exercise and Sport Research Group, Department of Physical Education and Sports, University of Valencia, 46010 Valencia, Spain

## Abstract

*Objectives*. To investigate core muscle activity, exercise preferences, and perceived exertion during two selected core exercises performed with elastic resistance versus a conventional training machine. *Methods*. 17 untrained men aged 26–67 years participated in surface electromyography (EMG) measurements of five core muscles during torso-twists performed from left to right with elastic resistance and in the machine, respectively. The order of the exercises was randomized and each exercise consisted of 3 repetitions performed at a 10 RM load. EMG amplitude was normalized (nEMG) to maximum voluntary isometric contraction (MVC). *Results*. A higher right erector spinae activity in the elastic exercise compared with the machine exercise (50% [95% CI 36–64] versus 32% [95% CI 18–46] nEMG) was found. By contrast, the machine exercise, compared with the elastic exercise, showed higher left external oblique activity (77% [95% CI 64–90] versus 54% [95% CI 40–67] nEMG). For the rectus abdominis, right external oblique, and left erector spinae muscles there were no significant differences. Furthermore, 76% preferred the torso-twist with elastic resistance over the machine exercise. Perceived exertion (Borg CR10) was not significantly different between machine (5.8 [95% CI 4.88–6.72]) and elastic exercise (5.7 [95% CI 4.81–6.59]). *Conclusion*. Torso-twists using elastic resistance showed higher activity of the erector spinae, whereas torso-twist in the machine resulted in higher activity of the external oblique. For the remaining core muscles the two training modalities induced similar muscular activation. In spite of similar perceived exertion the majority of the participants preferred the exercise using elastic resistance.

## 1. Introduction

With a lifetime prevalence ranging from 11 to 84% of the population, low back pain (LBP) is considered one of the major important health problems in the modern world [1]. Moreover, LBP has been associated with reduced work ability, lost productivity, prolonged sickness absence, and increased risk of not returning to work [2, 3] and hence poses a great socioeconomic burden.

The effect of physical activity regarding the prevention of LBP is still controversial [4–9]. Although conflicting data exists [10], most systematic reviews find that physical activity levels are not associated with or predictive of LPB but also serve to highlight the fact that comparisons between heterogeneous studies are difficult [5, 11]. These conflicting results highlight that LBP consists of a broad array of individual biopsychosocial factors and therefore most likely requires complex multidisciplinary rehabilitation in order to achieve success [12–14]. However, some studies indicate that core muscle function may be one of the pieces of the puzzle [15–18]. For example, isometric trunk extensor endurance has been shown to be predictive [15] and indicative [16] of LBP. In addition, trunk muscle endurance and the ratio between trunk flexion and extension strength have been shown to predict future LBP [17]. Furthermore, a number of recent randomized controlled trials (RCT) have shown that 10–20 weeks of localized physical training reduce musculoskeletal pain in various job groups [18–20]. Therefore, based on the potential negative effect of strength and endurance deficits of core muscles in regard to LBP, methods to effectively train these muscles are warranted.

The core muscles can be strengthened using specific exercise machines in a gym. However, previous research indicates that factors such as time and equipment accessibility play an important role in adherence to physical exercise [19]. Therefore, efficient core muscle exercises that can be performed outside the gym, for example, at the workplace, at home, or in limited rehabilitation facilities, are needed. Exercises utilizing elastic tubing have been tested for effectiveness in healthy individuals as well as in individuals with musculoskeletal pain [4, 21–23]. A recent study by Andersen et al. [24] showed comparable high levels of upper extremity muscle activation during resistance exercises using dumbbells and elastic tubing in healthy subjects, indicating that elastic tubing may be used as an effective user-friendly alternative to conventional exercises. However, it is currently unknown how effective an exercise modality elastic resistance is for the core muscles compared with more traditional resistance training in machines.

This study evaluates EMG activity during trunk rotation exercises using elastic resistance compared with a conventional training machine. In addition, this study compares perceived exertion, exercise duration, and participant preference, in relation to exercise modality. Based on previous research from our group comparing these exercise modalities [21, 23, 25], we tested the null hypothesis of no difference.

## 2. Methods

### 2.1. Subjects

Seventeen healthy and untrained (e.g., not engaged in strength training for the past 12 months) men were recruited from a large workplace with various job tasks in Copenhagen, Denmark. Inclusion criteria furthermore included being able to perform the exercises without issues or pain. Exclusion criteria were blood pressure above 160/100, disc prolapse, or serious chronic disease. Participants visited the laboratory on two separate occasions, separated by one week: on the first occasion the participants were habituated to the exercises, and on the second occasion the participants were tested with the aim of comparing the two exercise modalities.  Table 1 shows participant demographics. All participants were informed about the purpose and content of the project and gave written informed consent. The study conformed to The Declaration of Helsinki and was approved by the Local Ethical Committee (H-3-2010-062).

### 2.2. Maximal Voluntary Isometric Contraction (MVC)

Prior to the dynamic exercises, isometric MVCs were performed during trunk flexion and extension to induce a maximal EMG response of the tested muscles. Two isometric MVCs, separated by 2 min. of rest, were performed for each muscle, and the trial with the highest EMG was used for normalization of the peak EMG obtained during the resistance exercises. Subjects were instructed to gradually increase muscle contraction force towards maximum over a period of two seconds, sustain the MVC for three seconds, and then slowly decrease the force again [21–23]. Strong and standardized verbal encouragement was given during all trials.

### 2.3. Exercise Equipment, Description, and Preference

We used two different types of training equipment: (1) elastic tubing (TheraBand, Akron, Ohio, US) with resistance ranging from light to very heavy (red, green, blue, black, and silver) and (2) a lateral ab-crunch machine with loads ranging from 10 to 200 kg (horizontal seated ab-crunch, Technogym, Gambettola, Italy).

**Figure 1:**
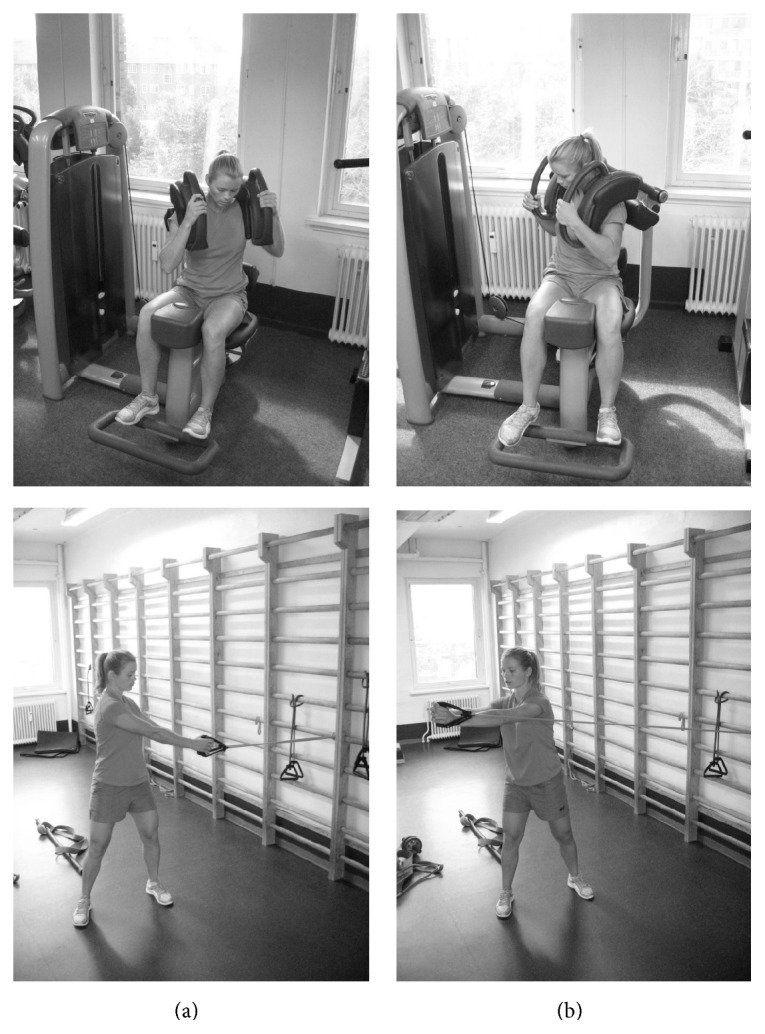
Illustration of the torso-twist in machine (top) and the torso-twist with elastic resistance (bottom). (a) and (b) indicate starting and ending position, respectively.

A week prior to testing the participants underwent familiarization of the exercises and identified their individual 10 repetition maximum (10 RM). Immediately after each set of exercise during this session, the Borg CR10 scale was used to rate perceived exertion [26]. Previous research from our group has found a strong correlation between nEMG and perceived loading on the Borg CR10 scale [21]. On the day of EMG measurements, participants warmed up with submaximal loads and subsequently performed three consecutive repetitions using the 10 RM loads, to avoid fatigue. All exercises were performed in a controlled manner, and the participants were not instructed to follow a fixed tempo during the repetitions. The order of the exercises was randomized for each subject, and the rest period between exercises was five minutes. The exercises are described below and shown in Figure 1.

*Torso-Twists with Elastic Resistance*. The participant stood with feet shoulder-width apart in a direction parallel to a wooden rib where the elastic band was attached. In the starting position with the trunk rotated to the left and the arms positioned horizontally and extended, the elastic tube was prestretched to twice its resting length (Figure 1). The participants were then asked to twist their torso from left to right, hereby increasing the elastic resistance. During the motion, the participants' feet, legs, and hip stayed stationary as the trunk rotated. One repetition was successfully completed when the participant's arms and torso returned to the starting position.

*Torso-Twist in Machine*. The participants were seated in the torso-twist machine rotated to the left, with the feet behind the ankle rollers and the hands holding the handles at shoulder level (Figure 1). They were asked to twist the body in a controlled motion from left to right, against resistance. When maximal torso rotation was reached, the participant returned to the starting position in a controlled manner.

After completion of both exercises, participants were asked the question: “*If you had to train your abdominal muscles regularly, which of the two exercises would you then prefer*?”

### 2.4. EMG Signal Sampling and Analysis

EMG signals were recorded from 5 muscles of the trunk: rectus abdominis, left and right external obliques, and left and right erector spinae. A bipolar surface EMG configuration (Blue Sensor N-00-S, Ambu A/S, Ballerup, Denmark) and an interelectrode distance of 2 cm were used. Before affixing the electrodes, the skin of the respective area was prepared with scrubbing gel (Acqua gel, Meditec, Parma, Italy), to ensure an impedance less than 10 kΩ [22, 24, 27]. Electrode placement followed the SENIAM recommendations [28].

The EMG electrodes were connected directly to wireless probes that preamplified the signal (gain 400) and transmitted data in real time to a nearby 16-channel PC-interface receiver (TeleMyo DTS Telemetry, Noraxon, Arizona, USA). The dimension of the probes was 3.4 cm × 2.4 cm × 3.5 cm. The sampling rate was set to 1500 Hz with a bandwidth of 10–500 Hz to avoid aliasing. The resolution of the signals was 16 bits. The common mode rejection ratio was better than 100 dB.

During later analysis all raw EMG signals obtained during MVCs as well as during the exercises were digitally filtered, consisting of (1) high-pass filtering at 10 Hz and (2) a moving root-mean-square (RMS) filter of 500 ms. For each individual muscle, peak RMS EMG of the 3 repetitions was determined, and the average value of these 3 repetitions was then normalized to the maximal RMS EMG obtained during MVC [4, 22, 27].

### 2.5. Statistics

A linear mixed model (Proc Mixed, SAS version 9.3, SAS Institute, Cary, NC) was used to locate differences between exercises and muscles. Factors included in the model were* exercise *(elastic resistance and machine) and* muscle* (the 5 muscles), as well as* exercise by muscle *interaction. Normalized EMG (nEMG) was the dependent variable. Analyses were controlled for age. When a significant main effect was found relevant post hoc comparisons were made to locate differences. Values are reported as least square means (95% confidence interval) unless otherwise stated. *P* values < 0.05 were determined to be significant.

## 3. Results

### 3.1. Exercise Evaluation

A significant* muscle by exercise* interaction was observed (*P* < 0.05). Torso-twists with elastic resistance showed higher activity of the right erector spinae compared to torso-twist performed in machine (*P* < 0.05). By contrast, torso-twist in machine showed higher activity of the left external oblique compared to torso-twists with elastic resistance (*P* < 0.005). The values for the 5 muscles are reported in Table 2.

Further, there was a main effect on contraction time (exercise duration); that is, contraction time was significantly higher in torso-twists with elastic resistance compared with torso-twist in machine (3439 ± 280 versus 2986 ± 279 ms., resp., *P* < 0.05).

### 3.2. Exercise Preference and Perceived Exertion

76% (13 out of 17) of the participants preferred the exercise utilizing elastic tubing over the torso-twist in the conventional exercise machine. Furthermore, no differences between exercises were observed in perceived exertion (5.7 [95% CI 4.81–6.59] versus 5.8 [95% CI 4.88–6.72], *P* > 0.80).

## 4. Discussion

The main findings of this study are that the torso-twist in machine demonstrated higher muscle activity of the left external oblique muscle compared with the torso-twist performed with elastic resistance, whereas the elastic exercise showed higher activation of the right erector spinae muscle. The majority of the participants preferred the exercise performed with elastic resistance over the machine.

The present study indicates that including both exercises in a rehabilitation or training program could be beneficial, as they activate trunk extensors and flexors differently. The differences in activation would most likely be a result of the two different body positions (seated versus standing), as it is likely that the standing position would engage the postural muscles to a larger degree due to a less fixed hip position. In addition, the fact that the elastic exercise was performed with a greater lever arm would also increase the demand on the postural muscles.

We recently investigated muscle activity during seated ab-crunches performed in a machine and on a Swissball with elastic resistance [23]. In that study, we found a high activation (>71% nEMG) of the external obliques and a low activation (<20% nEMG) of the erector spinae, both comparable to the values obtained from the seated torso-twist in the current study. The fact that we report similar low activation of the erector spinae could indicate a decreased reliance of the back extensor muscles when performing both ab-crunches and trunk rotations in a seated position. On the other hand, trunk rotation during standing position seems to increase muscle activity of the back extensors, potentially as a way to maintain balance and stability. However, it is likely that the increased reliance of the erector spinae in a standing position is exercise-specific. For example, a study by Saeterbakken and Fimland [29] observed no differences in erector spinae muscle activity when comparing dumbbell presses in the seated versus standing position.

The fact that no differences in muscle activity were observed in the other measured muscles during the two exercises was in accordance with our initial hypothesis. This finding is in line with results from previous studies, which demonstrate the efficacy of the elastic resistance to generate similar muscle activity compared to free-weight- [24, 25] and machine-based exercises [30]. Because there were similar levels of muscle activity in these muscles, it is likely that any potential strength gains over time would be similar as well [31].

When evaluating exercises for the core musculature and strengthening exercises in general, it is important to ascertain that the exercises are performed in accordance with the desired intensity and goal [32]. The fact that the majority of the selected core muscles during the two exercises did not reach nEMG values above 55% of isometric MVC would indicate a possible role for these exercises as an initial part of a rehabilitation program more so than as a part of a training protocol focusing on maximal strength. In this regard, the importance of stimulating the core muscles through low-intensity training in order to avoid any potential muscle recruitment imbalances that consequently may lead to movement dysfunction and injury has been highlighted in the literature [33]. In addition, it has been suggested that muscle endurance of the trunk extensors is important to prevent low back pain [34]. These notions further validate the use of low-intensity strength training in a rehabilitation setting, targeting selected core muscles for efficient integration in complex whole-body exercises.

In addition to the importance of exercise intensity and specificity, adherence to exercise is essential for success in rehabilitation and/or training. In the current study, the majority of participants preferred the elastic torso-twist over torso-twist in machine. Notably, participants rated perceived exertion identical between the two exercises (5.7 and 5.8 for the elastic resistance and machine, resp.). These results are therefore in accordance with the existing literature [21, 22, 27], highlighting the use of elastic resistance as a user-friendly alternative to machine-based core exercises, especially in a rehabilitation setting where compliance and low-cost alternatives are needed. Furthermore, without the participants being instructed to differentiate, the exercise utilizing elastic resistance showed an increased contraction time compared to the machine. This finding is likely the result of an inherent greater range of motion in the exercise utilizing elastic resistance. This may result in increased hypertrophy over time, as greater time under tension would cause fatigue across a larger spectrum of muscle fibers [35].

Limitations of this study include the fact that only core muscles were measured during the exercises. Taking into account the relative low activation of the selected core muscles combined with the perceived exertion rated as moderate/hard, this indicates a very high likelihood of other muscles participating in the exercises as prime movers. It is possible that during both exercises in this study (and especially in the standing position) the musculature of the upper arms, shoulders, and muscles stabilizing the spine were highly active, which is why recordings of the upper-body/extremity muscles would have been of interest. It is therefore important to focus on the core musculature as prime movers and not the upper extremities, when giving instructions for these specific exercises. Lastly, the present study was conducted with healthy subjects, so the clinical applications for patient groups remain unknown.

## 5. Conclusion

Torso-twists using elastic resistance showed a higher activation of the erector spinae, whereas torso-twist in the machine resulted in a higher activation of the external oblique. For the remaining core muscles the two training modalities induced similar muscular activation. In addition, even though the perceived exertion was rated identical, the majority of the participants preferred the exercise using elastic resistance. Although choosing only one core exercise might not be sufficient, elastic resistance generally has the potential to provide similar muscular adaptations as core exercises performed in the machine and may especially be useful in a rehabilitation setting. However, the clinical application of utilizing elastic resistance is warranted.

## Figures and Tables

**Table 1:** Demographics. Values are mean (SD).

Demographics	
*N*	17
Age, yrs	41 (13.6)
Height, cm	179 (6.8)
Weight, kg	78 (8.9)
BMI	24 (1.9)

**Table 2:** Normalized EMG (nEMG) values of the 5 selected muscles during torso-twist in machine and using elastic resistance, respectively. Values are presented as least square means (LSM) and 95% confidence interval (95% CI).

Muscle	Elastic	Machine	Mean diff. (95% CI)	*P* value
	Mean (95% CI)	Mean (95% CI)		
Rectus abdominis	10 (−3 to 24)	16 (3 to 29)	−5.6 (−20 to 9)	0.45
External obliques (left)	54 (40 to 67)	77 (64 to 90)	−23 (−38 to −9)	0.0018
External obliques (right)	47 (34 to 60)	41 (28 to 54)	6.2 (−8 to 20)	0.39
Erector spinae (left)	24 (10 to 37)	18 (4 to 32)	5.9 (−10 to 22)	0.47
Erector spinae (right)	50 (36 to 64)	32 (18 to 46)	17.7 (1.6 to 34)	0.03
